# Supplementary material for: Enhanced Neural Empathic Responses in Patients with Spino-Bulbar Muscular Atrophy: An Electrophysiological Study
Source: Brain Sci. 2020 Dec 24;11(1):16. doi: 10.3390/brainsci11010016 (PMC7824338; doi:10.3390/brainsci11010016)
Supplement: Supplementary file 1 [file brainsci-11-00016-s001.pdf]

Supplementary Table 1. Scores and comparisons of neuropsychological screenings and explicit dispositional empathy assessments

Test	Patients		Controls		Mann-Whitney		Cohen's *d*
	M	*SD*	*M*	*SD*	*U*	p_{corr}	
Forward Digit Span	5.78	1.35	5.89	0.76	172	.70	.10
Backwards Digit Span	4.50	1.10	4.56	0.92	170	.70	.05
Phonemic Fluency Test	40.39	11.90	46.67	13.42	210	.70	.50
Prose Memory Test	14.84	1.71	13.92	1.42	91.5	.99	.59
Trail Making Test B-A	39.67	18.29	34.94	10.78	134.5	.95	.31
Mental Rotation Test	6.72	4.90	5.56	2.23	155.5	.88	.31
IRI Empathic Concern	26.63	4.51	26.22	2.65	115	.70	.11
IRI Perspective Taking	22.83	3.91	23.00	3.77	174.50	.70	.04

Notes. The table reports the means and the standard deviations of all neuropsychological screenings and explicit dispositional empathy assessments for both patients and control participants. Trail Making Test B-A is the difference between the B and A subtests. The comparison between patients' and controls' scores are reported through Mann-Whitney U tests and Cohen's *d* effect size (details in manuscript).

Cohen's effect size, small: $d = 0.2$, medium: $d = 0.5$, large: $d = 0.8$ [1].

1. Cohen, J. *Statistical Power Analysis for the Behavioral Sciences*; Routledge Academic: New York, NY, 1988
